# Chemical Composition, Diuretic, and Antityrosinase Activity of Traditionally Used Romanian *Cerasorum stipites*

**DOI:** 10.3389/fphar.2021.647947

**Published:** 2021-05-11

**Authors:** Mihai Babotă, Oliviu Voştinaru, Ramona Păltinean, Cosmin Mihali, Maria Inês Dias, Lillian Barros, Isabel C. F. R. Ferreira, Andrei Mocan, Ovidiu Crişan, Cristina Nicula, Gianina Crişan

**Affiliations:** ^1^ Department of Pharmaceutical Botany, “Iuliu Haţieganu” University of Medicine and Pharmacy, Cluj-Napoca, Romania; ^2^ Department of Pharmacology, Physiology and Physiopathology, “Iuliu Haţieganu” University of Medicine and Pharmacy, Cluj-Napoca, Romania; ^3^ Centro de Investigação de Montanha (CIMO), Instituto Politécnico de Bragança, Campus de Santa Apolónia, Bragança, Portugal; ^4^ Laboratory of Chromatography, ICHAT, University of Agricultural Sciences and Veterinary Medicine Cluj-Napoca, Cluj-Napoca, Romania; ^5^ Department of Organic Chemistry, “Iuliu Haţieganu” University of Medicine and Pharmacy, Cluj-Napoca, Romania; ^6^ Department of Ophthalmology, “Iuliu Haţieganu” University of Medicine and Pharmacy, Cluj-Napoca, Romania

**Keywords:** *Cerasus sp.*, cherry stems, diuretic activity, polyphenols, by-products

## Abstract

Cherry stems (CS) represent a by-product intensively used in Eastern European countries as a traditional remedy for urinary tract disorders. Ethnopharmacological evidences sustain the use of CS as aqueous preparations (infusion and decoction), but few data were previously reported about phytochemical profile and pharmacological potential of CS hydroalcoholic extracts. In this regard, we aimed to evaluate the phenolic profile, *in vitro* antioxidant and tyrosinase inhibitory potential, and *in vivo* diuretic activity of 70% hydroethanolic cherry stems extract and cherry stems decoction (CSD). LC-DAD-ESI/MS^n^ analysis revealed the presence of flavonoid-type compounds as main constituents for both preparations, especially flavanones (naringenin glycosides). Antioxidant activity evaluated through DPPH, ABTS, and FRAP methods was superior for cherry stems extract, probably due to the presence of phenolic-derived compounds in higher amounts than CSD. On the other hand, tyrosinase inhibitory potential and diuretic effect exerted by CSD were stronger, highlighting that other types of hydrophilic secondary metabolites are responsible for this bioactivity. Overall, our findings indicate that CS preparations could be used as promising mild diuretic agents and encourage further investigations regarding the correlation between their chemical composition and bioactive potential.

## Introduction

In last decades, the importance of plant-derived bioactive compounds was intensively studied, highlighting their impact on human health as modulators of metabolic pathways and processes involved in the development of different pathological conditions. Even though dietary intake can provide high amounts of plant-derived secondary metabolites, food habits and nutritional quality of our meals upset the balance between the real need and consumption of these compounds. According to Bailey (2020), more than 50% of U.S. adult population use dietary supplements; a study on E.U. food supplement market conducted by European Commission estimates that 50% of these products are based on vitamins and minerals, 43% food supplements containing other substances, and 7% tonics and bottled nutritional drinks (classified as OTC products or marketed as functional foods) (European Advisory Services (EAS), 2007). Hence, the interest for this type of products is in a continuous and exponential increase, explaining the actual trends in the research for novel and sustainable resources for food supplements industry.

By-products resulted from the processing of different herbal resources are intensively promoted as promising sources of bioactive compounds, being recognized as cheap, eco-friendly, and sustainable alternatives for standard raw materials (Fierascu et al., 2020). Moreover, recent studies have proven that high amounts of secondary metabolites can be concentrated in plant by-products; for example, it was shown that an important fraction from total polyphenolic content of several by-products from food industry can be found as bound form, namely non-extractable polyphenols (NEP), which exerts significant antitumor, antioxidant, and hypocholesterolemiant properties (Dzah et al., 2020). Therefore, it can be assumed that one of the big goals for the future is the valorification of more and more by-products as main sources of phytochemicals with health-related properties.

Cherry stems (CS) are one of the main by-products obtained after the harvesting and processing of sweet cherries (*Prunus avium*) and sour cherries (*Prunus cerasus*). Even though these are generally recognized as a waste for food industry, the folk medicine recommends them as a traditional herbal remedy, especially for its diuretic and sedative properties; the herbal drug is known by its Latin botanical name (*Cerasorum stipites*), being used as infusion or decoction (Bastos et al., 2015; Aires et al., 2017; Švarc-Gajić et al., 2018; Nastić et al., 2020; Peixoto et al., 2020). According to the Committee on Herbal Medicinal Products (HMPC) of European Medicines Agency, CS can be used single or as herbal tea combinations with other herbal substances used in the therapeutic area for “urinary tract disorders,” generically named *Species diureticae* (Länger, 2017). In Central and Eastern European countries, especially in Romania, based on ethnopharmacological recommendations, the herbal product is recognized as a popular remedy in the adjuvant treatment of kidney stones, mild urinary tract infections, edema, and hypertension. In *Encyclopedia of Romanian ethnobotany*, Butura *et al.* describe the use of CS as infusions or decoctions in intra-Carpathic areas in the treatment of kidney disorders; different traditional recipes for decoctions were reported, obtained from both stems and crushed stones from cherries or herbal mixtures including aerial parts of horsetail (*Equisetum arvense*), leaf buds of mountain pine (*Pinus mugo*), and corn stigmas (*Zea mays*) (Butura, 1979). Being one of the most popular herbal products with diuretic properties in Romanian folk medicine, pharmacies and markets sell CS-containing products conditioned as teas or capsules, recommended as adjuvant therapy in kidney stones, cystitis, or obesity (Pârvu, 2006).

Few phytochemical and pharmacological investigations were conducted on CS. Most of the studies available on this topic are from last 5 to 10 years, being focused both on qualitative and quantitative evaluation of the main constituents in the extracts and the assessment of their potential bioactivities. In a comparative study between fruits and stems focused on hydrophilic compounds profile, Bastos *et al*. showed that stems are rich in citric, malic, and oxalic acids (Bastos et al., 2015). In the same study, phenolic profile of hydroalcoholic extracts obtained from stems was evaluated; sakuranetin-5-*O*-glucoside, catechin, naringenin-7-*O*-glucoside, and aromadendrin-7-*O*-hexoside were quantified as main compounds. Other studies revealed the presence of hydroxycinnamic acids (*cis*-3-*O*-*p*-coumaroylquinic acid, chlorogenic acid, and *trans*-3-*O*-*p*-coumaroylquinic acid): quercetin and sinapic acid in high amounts (Demir et al., 2020; Peixoto et al., 2020). In this regard, corroborated with the occurrence of these compounds, several *in vitro* bioactivities were proven for CS extracts: antioxidant, antitumor, antidiabetic, and antibacterial (Bastos et al., 2015; Aires et al., 2017; Demir et al., 2020; Peixoto et al., 2020).

Except for the *in vitro* antibacterial effect on *E. coli* strains, few studies were focused on the evaluation of the real benefits of CS in the treatment of urinary tract disorders based on pharmacological investigation (Aires et al., 2017). Hence, the present study aimed to evaluate phenolic profile and pharmacological properties of two different types of extracts obtained from CS (hydroethanolic and aqueous decoction). LC-DAD-ESI/MS^n^ technique was employed for both qualitative and quantitative analysis of several phenolic compounds, followed by *in vitro* evaluation of antioxidant and tyrosinase inhibitory activities. *In vivo* diuretic potential of the extracts was tested using a rodent model previously established.

## Materials and Method

### Chemicals and Reagents

Acetonitrile (99.9%) was of HPLC grade from Fisher Scientific (Lisbon, Portugal). Phenolic compound standards (chlorogenic acid, ferulic acid, naringenin, *p*-coumaric acid, quercetin-3-O-glucoside, quercetin-3-O-rutinoside, and taxifolin) were from Extrasynthèse (Genay, France). Formic acid was purchased from Sigma-Aldrich (St. Louis, MO, United States). All other general laboratory reagents were purchased from Panreac Química S.L.U. (Barcelona, Spain). Water was treated by using a Milli-Q water purification system (TGI Pure Water Systems, Greenville, SC, United States). Ferric chloride; 6-hydroxy-2,5,7,8-tetramethylchromane-2-carboxylic acid (Trolox) (97%); diammonium 2,2′-azino-bis(3-ethylbenzothiazoline-6-sulfonate) (ABTS) (>98%); 2,2-diphenyl-1-(2,4,6-trinitrophenyl) hydrazine (DPPH); 2,4,6-tris (2-pyridyl)-s-triazine (TPTZ) (≥99%); dimethyl sulfoxide (DMSO) (≥99%); phosphate buffer, mushroom tyrosinase; 3,4-dihydroxy-l-phenylalanine (l-DOPA) (≥98%); and kojic acid were purchased from Sigma (Sigma-Aldrich Chemie GmbH, Schnelldorf, Germany). All other reagents used, including solvents, were of analytical grade.

### Plant Material

CS were purchased from a community pharmacy in Cluj-Napoca, Romania, as herbal tea conditioned *in toto* (raw material). Samples were ground to a fine powder using a laboratory mill, sieved and immediately subjected to extraction. The quality of samples was analyzed and confirmed after organoleptic and macroscopic control by Dr. Andrei Mocan from Department of Pharmaceutical Botany, Faculty of Pharmacy, “Iuliu Hatieganu” University of Medicine and Pharmacy (Cluj-Napoca, Romania).

### Extraction Procedure

*Maceration:* 100 g CS powder previously weighed and transferred in an Erlenmeyer flask was mixed with 500 ml of 70% (*v:v*) ethanol, being shaken and kept at room temperature in a dark place for 72 h. After filtration, the residue was re-extracted with 500 ml of solvent in the same condition for other 6 days. The extracts were reunited in a round-bottom flask and the alcohol was evaporated under reduced pressure. The aqueous suspension obtained was further lyophilized; the dry CS extract (CSE) was being kept in a desiccator at room temperature until analysis.

*Decoction:* 1 L of boiling water was added over 100 g CS powder previously weighed in an Erlenmeyer flask, the mixture being maintained at 100 °C on an electric hob under continuous stirring. The aqueous extract was hot filtered, cooled at room temperature, and lyophilized, obtaining a dry CS decoction (CSD). This extract was kept in similar conditions with CSE until further analysis.

### LC-DAD-ESI/MS Analysis of Phenolic Compounds

The phenolic profile was determined by LC-DAD-ESI/MS^n^ (Dionex Ultimate 3000 UHPLC, Thermo Scientific, San Jose, CA, United States). These compounds were separated and identified using a method previously described (Bessada et al., 2016). The obtained extracts were redissolved at a concentration of 10 mg/ml with methanol: water (80:20, *v/v*) mixture. A double online detection was performed using a DAD (280, 330, and 370 nm as preferred wavelengths) and a mass spectrometer (MS). The MS detection was performed in negative mode, using a Linear Ion Trap LTQ XL mass spectrometer (Thermo Finnigan, San Jose, CA, United States) equipped with an ESI source.

The identification of the phenolic compounds was performed based on their chromatographic behavior and UV-Vis and mass spectra by comparison with standard compounds, when available, and data reported in the literature giving a tentative identification. Data acquisition was carried out with Xcalibur^®^ data system (Thermo Finnigan, San Jose, CA, United States). For quantitative analysis, a calibration curve for each available phenolic standard was constructed based on the UV-Vis signal. For the identified phenolic compounds for which a commercial standard was not available, the quantification was performed through the calibration curve of the most similar available standard. The results were expressed as mg/g of extract.

### Total Phenolic Content (TPC) and Total Flavonoid Content (TFC)

Samples redissolved in 70% ethanol with 5% DMSO at concentration of 1 mg/ml were evaluated using protocols previously described by Mocan et al. (2017). For TPC determination, samples (20 µL) were mixed with diluted Folin–Ciocalteu reagent (1:9, *v/v*) (100 µL) and shaken vigorously. After 3 min, Na_2_CO_3_ solution (1%) (80 µL) was added and the sample absorbance was read at 760 nm after 30 min incubation at room temperature. The total phenolic content was expressed as milligrams of gallic acid equivalents (mg GAE/g extract). In TFC assay, samples (100 µL) were mixed with 2% aluminum trichloride methanolic solution (100 µL). The sample absorbance was read at 415 nm after 10 min incubation at room temperature. Rutin was used as a reference standard and the total flavonoid content was expressed as milligrams of rutin equivalents (mg RE/g extract).

### Preliminary Biochemical Assessment of Antioxidant Potential

#### Antioxidant Assays

The antioxidant potential of CSE and CSD was tested through three complementary methods (DPPH, ABTS, and FRAP), the protocols being previously described by Mocan et al. (2017), Babotă et al. (2018), and Rusu et al. (2018). Samples were redissolved in 70% ethanol with 5% DMSO obtaining 1 mg/ml concentration; these were further analyzed using a SPECTROstar^®^ nano multi-detection microplate reader with 96-well plates (BMG Labtech, Ortenberg, Germany).

*DPPH (1,1-diphenyl-2-picrylhydrazyl) radical scavenging assay:* Samples (30 μL) were mixed with a 0.004% methanol solution of DPPH (270 μL). The absorbance was read at 517 nm after 30 min incubation at room temperature in the dark. DPPH radical scavenging activity was expressed as milligrams of Trolox equivalents (mg TE/g extract).

*ABTS (2,2′-azino-bis(3-ethylbenzothiazoline)-6-sulfonic acid) radical scavenging assay:* Briefly, ABTS^+^ was produced directly by reacting 7 mM ABTS solution with 2.45 mM potassium persulfate and allowing the mixture to stand for 12–16 in the dark at room temperature. Prior to beginning the assay, ABTS solution was diluted with methanol to an absorbance of 0.700 ± 0.02 at 734 nm. Samples (20 μL) were added to ABTS solution (10 mM and 200 μL) and mixed. The sample absorbance was read at 734 nm after 30 min incubation at room temperature. The ABTS radical scavenging activity was expressed as millimoles of Trolox equivalents (mmol TE/g extract).

*FRAP (ferric reducing antioxidant power) activity assay:* Samples (25 μL) were added to premixed FRAP reagent (175 μL) containing acetate buffer (0.3 M, pH 3.6); 2,4,6-tris(2-pyridyl)-*s*-triazine (TPTZ) (10 mM) in 40 mM HCl; and ferric chloride (20 mM) in a ratio of 10:1:1 (*v/v/v*). Then, the sample absorbance was read at 593 nm after 30 min incubation at room temperature. FRAP activity was expressed as milligrams of Trolox equivalents (mg TE/g extract).

### Biological Activities Evaluation

#### Tyrosinase Inhibition

Sample solution (10 mg/ml, 25 μL) was mixed with tyrosinase solution (40 μL, Sigma) and phosphate buffer (100 μL, pH 6.8) in a 96-well microplate and incubated for 15 min at 25 °C. The reaction was then initiated with the addition of l-DOPA (40 μL, Sigma). Similarly, a blank was prepared by adding sample solution to all reaction reagents without enzyme (tyrosinase) solution. The sample and blank absorbances were read at 492 nm after 10 min incubation at 25 °C. The absorbance of the blank was subtracted from that of the sample and the tyrosinase inhibitory activity was expressed as inhibition percentage (IC_50_) (mg/ml), calculated for each sample and kojik acid (positive control) (Uysal et al., 2017).

#### *In vivo* Studies on Diuretic Effect

*Animals:* Forty-eight adult male Charles River Wistar rats (Crl:WI) with a medium weight of 151 ± 8 g were obtained from the Practical Skills and Experimental Medicine Center of the “Iuliu Haţieganu” University of Medicine and Pharmacy, Cluj-Napoca, Romania. The rats were housed in polycarbonate cages (Tecniplast, Italy) and maintained under standard conditions (22 ± 2 °C, a relative humidity of 45 ± 10%, and 12:12-h light: dark cycle). The animals had free access to standard pelleted food (Cantacuzino Institute, Bucharest, Romania) and filtered water throughout the experiment, except for the day when the test substances were administered. All experimental protocols were approved by the Ethics Committee of the University (Approval No. 168/April 7, 2017) and were conducted in accordance with the EU Directive 86/609/EEC, which regulates the use of laboratory animals for scientific research.

*Diuretic and saluretic effects:* Diuretic and saluretic effects of CSE and CSD were tested by a method using isotonic saline solution in order to achieve an optimal hydration (Kau et al., 1984). Eight groups of Crl:WI rats (*n* = 6) were used. The negative control group of rats was treated orally by gavage only with 25 ml/kg isotonic saline solution (Braun, Germany), while the positive control group was treated orally with 10 mg/kg furosemide (Zentiva, Romania), a reference diuretic drug dissolved also in a volume of 25 ml/kg isotonic saline solution. Three groups of rats were treated orally with 125, 250, and 500 mg/kg CSE dispersed in the same volume of 25 ml/kg isotonic saline solution, respectively, while other three groups of rats received also by oral route 125, 250, and 500 mg/kg CSD dispersed in 25 ml/kg isotonic saline solution.

After the substance administrations, rats were individually placed in metabolic cages. The urine output (ml) was recorded for each animal at two time intervals: 5 and 24 h after the administration of a single dose from the tested substances (Zhang et al., 2010). Diuretic action was calculated at 24 h as the ratio of urine output in test groups to urine output in negative control group. Diuretic activity was calculated at 24 h as the ratio of urine output in test groups to urine output of the positive control (reference) group.

Additionally, the saluretic effect of CSE and CSD was investigated. The urinary concentration of sodium and potassium ions (U_Na_ and U_K_) was twice determined in the collected urine samples, 5 and 24 h after the substance administration, by a potentiometric method with selective electrodes, using a VITROS 250 Chemistry System automatic analyzer (Johnson and Johnson Clinical Diagnostic), being expressed in mEq/kg (Pǎltinean et al., 2017). Furthermore, at 24 h, blood samples were obtained from all animals by retro-orbital sinus puncture under ketamine/xylazine anesthesia and serum concentration of sodium ions (S_Na_) was determined by the same potentiometric method. Creatinine was spectrophotometrically determined in the serum and urine at 670 nm, with the VITROS 250 Chemistry System automatic analyzer, using a reaction which formed a triaryl imidazole leuco dye. Fractional excretion of sodium ions (FE_Na_) was calculated with the formula

$$FE_{\mathrm{Na}}=\;\frac{U_{\mathrm{Na}}\;\times \;CR_{S}}{S_{\mathrm{Na}}\;\times \;CR_{U}}\;\times \;100$$

where U_Na_ is the urine concentration of sodium ions, S_Na_ is the serum concentration of sodium ions, CR_S_ is the serum concentration of creatinine, and CR_U_ the urine concentration of creatinine (Păltinean et al., 2017).

### Statistical Analysis

Data were expressed as mean values ± SD and were statistically analyzed by the one-way ANOVA method. The differences between the treated groups and the negative control group were evaluated by Dunnett’s t-test using GraphPad Prism six software (GraphPad Software, United States); p values <0.05 being considered statistically significant.

## Results and Discussion

### Chemical Profile of the Extracts

#### Total Phenolic and Total Flavonoidic Content

Evaluation of TPC using the Folin–Ciocalteu method revealed a clear difference between the two extracts based on extraction parameters applied (Table 1). The 70% ethanol increased the extraction yield of phenolic compounds in CSE (TPC = 37.63 ± 2.75 mg GAE/g extract) by almost 50% in comparison with CSD (TPC = 19.11 ± 1.52 mg GAE/g extract). A similar trend was observed for TPC, the values obtained being 12.03 ± 0.72 mg QE/g extract for CSE and 5.34 ± 0.23 mg QE/g extract for CSD. Our results are in-line with those previously reported in other studies. The evaluation of TPC in a hydroalcoholic extract obtained after an optimized process by Demir et al. revealed that TPC value was 26.60 mg GAE/g extract (in comparison with predicted value, 26.1 mg GAE/g extract), optimal conditions being 35% (*v/v*) ethanol percentage, 79°C, and 119 min; for the same extract, the TFC value was 2.26 mg QE/g extract (Demir et al., 2020). On the other hand, aqueous extracts confirmed the presence of lower concentrations for both TPC and TFC - 24.31 ± 0.86 to 39.54 ± 2.10 μg GAE/mL, respectively, 15.67 ± 0.68 to 23.17 ± 1.23 μg CE (catechin equivalents)/mL for infusions (Peixoto et al., 2020). This can be correlated with low hydrophilicity of chemical components from CS and encourage the use of ethanol–water mixtures as extraction solvents in order to obtain phenolics-enriched extracts.

**TABLE 1:** Total phenolic and flavonoid content, DPPH and ABTS scavenging capacity, and ferric reducing ability of plasma (FRAP) of the extracts of CSE and CSD (values expressed are means ± S.D. of three parallel measurements, *p* < 0.05).

Probe ID	TPC (mg GAE/g extract)	TFC (mg QE/g extract)	DPPH scavenging (mmol TE/g extract)	ABTS scavenging (mg TE/g extract)	FRAP (mg TE/g extract)
CSE	37.63 ± 2.75	12.03 ± 0.72	30.02 ± 0.58	107.14 ± 1.43	111.87 ± 4.14
CSD	19.11 ± 1.52	5.34 ± 0.23	14.32 ± 2.00	55.65 ± 3.62	61.07 ± 2.83

#### LC-DAD/ESI–MS^2^ Analysis of Phenolic Profile

The solvents used and the extraction procedure play a significant role on the chemical profile of the extracts and their bioactive potential. In this regard, an LC-DAD/ESI–MS^2^ technique was employed to evaluate the phenolic compounds present in CSE and CSD. Data of the retention time, *λ*_max_, pseudomolecular ion, main fragment ions in MS^2^, and tentative identification of phenolic acid and flavonoid derivatives are presented in Table 2. Five major types of phenolic compounds were found in CSE and CSD: *phenolic acids* (*p*-coumaroyl (1, 5, and 6), caffeoyl (2, 3), and feruloyl 12) derivatives), *flavanones* (4, 7–9, 13–15, 18–20, and 24–27), *flavones* (10, 16, 21, and 22), *flavanols* (11), and *flavonols* (17 and 23).

**TABLE 2:** Retention time (Rt), wavelengths of maximum absorption in the visible region (*λ*_max_), mass spectral data, tentative identification and quantification (mg/g of extract) of the phenolic compounds present in CSE and CSD.

Peak	Rt (min)	*λ*_max (nm)_	Molecular ion [M-H]^-^ (*m/z*)	MS^2^ (*m/z*)	Tentative identification	Quantification		*t-*Students test *p*-value
						*CSE*	*CSD*	
1	6.1	310	337	191 (100), 173 (15), 163 (20), 155 (5)	3-*p*-Coumarouylquinic acid^(A)^	0.379 ± 0.004	nd	–
2	6.5	322	353	191 (45), 179 (68), 173 (100), 161 (5), 135 (10)	4-*O*-Caffeoylquinic acid^(B)^	0.69 ± 0.01	0.657 ± 0.002	<0.001
3	7.2	324	353	191 (100), 179 (13), 173 (5), 161 (5), 135 (5)	5-*O*-Caffeoylquinic acid^(B)^	1.15 ± 0.01	0.290 ± 0.001	<0.001
4	7.6	287,316 sh	401	269 (100)	Naringenin-*O*-pentoside^(C)^	Tr	nd	–
5	8.6	310	325	163 (100)	*p*-Coumaric acid hexoside^(A)^	0.692 ± 0.001	0.238 ± 0.003	<0.001
6	10.0	310	325	163 (100)	*p*-Coumaric acid hexoside^(A)^	0.221 ± 0.004	0.120 ± 0.002	<0.001
7	11.2	283,320 sh	449	287 (100)	Aromadendrin-*O*-hexoside isomer 1^(C)^	1.27 ± 0.02	0.46 ± 0.01	<0.001
8	11.7	282,322 sh	449	287 (100)	Aromadendrin-*O*-hexoside isomer 2^(C)^	0.31 ± 0.01	0.37 ± 0.01	<0.001
9	12.7	283,320 sh	449	287 (100)	Aromadendrin-*O*-hexoside isomer 3^(C)^	0.019 ± 0.001	0.054 ± 0.001	<0.001
10	13.8	352	609	301 (100)	Quercetin-deoxyhexoside hexoside^(D)^	Tr	0.027 ± 0.001	–
11	14.2	284,339 sh	465	303 (100)	Taxifolin-3-*O*-glucoside^(E)^	0.47 ± 0.01	0.125 ± 0.001	<0.001
12	15.2	320	355	193 (100), 149 (5), 134 (5)	Ferulic acid hexoside^(F)^	0.133 ± 0.002	0.053 ± 0.001	<0.001
13	15.9	282,325 sh	433	271 (100)	Naringenin-*O*-hexoside isomer 1^(C)^	0.29 ± 0.01	0.139 ± 0.002	<0.001
14	16.3	283	449	287 (100)	Aromadendrin-*O*-hexoside isomer 4^(C)^	0.077 ± 0.001	0.046 ± 0.001	<0.001
15	16.9	283,325 sh	433	271 (100)	Naringenin-7-*O*-glucoside ^(C)^	4.57 ± 0.09	0.71 ± 0.01	<0.001
16	17.9	353	609	301 (100)	Quercetin-3-*O*-rutinoside^(D)^	1.01 ± 0.03	0.404 ± 0.003	<0.001
17	19.1	351	463	301 (100)	Quercetin-3-*O*-glucose^(G)^	0.693 ± 0.004	0.14 ± 0.01	<0.001
18	19.9	260,330 sh	431	269 (100)	Genistein-7-*O*-glucoside^(C)^	0.0588 ± 0.002	0.0061 ± 0.0001	<0.001
19	20.4	281,330 sh	431	269 (100)	Genistein-*O*-hexoside^(C)^	Tr	0.043 ± 0.002	–
20	21.0	283,327 sh	433	271 (100)	Naringenin-*O*-hexoside isomer 2^(C)^	0.60 ± 0.01	0.078 ± 0.001	<0.001
21	21.2	340	593	285 (100)	Kaempferol-3-*O*-rhamnoside^(D)^	Tr	0.033 ± 0.001	–
22	22.2	350	623	315 (100)	Isorhamnetin-3-*O*-rhamnoside ^(D)^	Tr	Tr	–
23	22.6	342	447	285 (100)	Kaempferol-3-*O*-glucoside^(G)^	0.520 ± 0.002	0.108 ± 0.003	<0.001
24	24.2	280,323 sh	447	285 (100), 270 (5)	Dihydrowogonin 7-*O*-glucoside/sakuranetin 5-*O*-glucoside^(C)^	0.44 ± 0.02	0.025 ± 0.001	<0.001
25	26.9	255,320 sh	445	283 (100), 268 (20)	Methylgenistein hexoside^(C)^	0.95 ± 0.01	0.078 ± 0.001	<0.001
26	29.9	283,320 sh	417	255 (100)	Pinocembrin-7-*O*-glucoside^(C)^	Tr	Tr	–
27	31.9	286,335 sh	447	285 (100), 270 (5)	Dihydrowogonin 7-*O*-glucoside/sakuranetin 5-*O*-glucoside^(C)^	Tr	0.0059 ± 0.0001	–
Total phenolic compounds						**14.54 ± 0.12**	**4.205 ± 0.002**	<0.001

Nd, not detected; tr-trace amounts. Standard calibration curves: A—*p*-coumaric acid (*y* = 301950*x* + 6,966.7, *R* ^*2*^ = 0.9999, LOD = 0.68 μg/ml, and LOQ = 1.61 μg/ml); B—chlorogenic acid (*y =* 168823*x* – 161,172*; R* ^*2*^ = 0.9999, LOD = 0.20 μg/ml, and LOQ = 0.68 μg/ml); C—naringenin (*y* = 18,433*x* + 78,903, *R* ^*2*^ = 0.9998, LOD = 0.17 μg/ml,, and LOQ = 0.81 μg/ml); D—quercetin-3-*O*-rutinoside (*y* = 13,343*x* + 76,751, *R* ^*2*^ = 0.9998, LOD = 0.21 μg/ml, and LOQ = 0.71 μg/ml); E—taxifolin (*y* = 203766*x* – 208,383, *R* ^*2*^ = 1, LOD = 0.67 μg/ml, and LOQ = 2.02 μg/ml); F—ferulic acid (*y* = 633126*x* – 185,462, *R* ^*2*^ = 0.999, LOD = 0.20 μg/ml, and LOQ = 1.01 μg/ml); G—quercetin-3-*O*-glucoside (*y* = 34,843*x* – 160,173, *R* ^*2*^ = 0.9998, LOD = 0.21 μg/ml, and LOQ = 0.71 μg/ml).

With the exception of *p*-coumaroylquinic acid (compound 1), detected only in CSE−0.379 ± 0.004 mg/g, all phenolic acids were detected in both extracts, being found in higher concentrations in CSE than CSD. Compounds 1, 2, and 3 were identified as hydroxycinnamoyl derivatives, taking into account their fragmentation pattern and UV spectra at around *λ*_max_ = 314–330 nm. Compounds 1 [(M–H)^−^at *m/z* of 337], 2, and 3 [(M–H)^−^ at *m/z* of 353], all producing fragment ions with *m/z* 191, corresponding to the deprotonated quinic acid, so that they could be clearly identified as quinic acid derivatives containing a caffeic or coumaric acid units. Taking into account the following the hierarchal key developed by Clifford et al. (2003) and Clifford et al. (2006) for the identification of chlorogenic acid derivatives, these compounds were identified as 3-*p*-coumaroylquinic, 4-*O*-caffeoylquinic, and 5-*O*-caffeoylquinic acids. Compounds 5, 6 [(M–H)^−^at *m/z* of 325], and 12 [(M–H)^−^ at *m/z* of 355] were identified as coumaric acid hexoside and ferulic acid hexoside, all indicating the losses of a hexosyl moieties (Bastos et al., 2015; Bessada et al., 2016). The presence of phenolic acids in cherry fruits hydroalcoholic extracts was previously confirmed by Bastos et al. (2015) and Demir et al. (2020); *cis*-*p*-coumaroylquinic acid (0.56 ± 0.01 mg/g) and *trans*-*p*-coumaroylquinic acid (0.23 ± 0.02 mg/g) were reported, the amounts being comparable with our results obtained for determination of 3-*p*-coumaroylquinic acid in CSE (0.379 ± 0.004 mg/g). As we mentioned, this compound was identified only in CSE, probably due to hydroalcoholic medium, emphasizing the importance of the solvent for a better recovery of bioactive compounds.

The group of flavanones comprises the most representative compounds identified in CSE and CSD. Compounds 13, 15, and 20, showed the same pseudomolecular ion [M–H]^−^ at *m/z* 433, thus presented a different retention time, therefore, being tentatively identified as naringenin-*O*-hexoside isomers, despite that the position and nature of the sugar could not be identified for two of the isomers, thus compound 15 was assigned as naringenin-7-*O*-glucoside, which was quantified as the main component of CSE (4.57 ± 0.09 mg/g). Compound 4 [(M–H)^−^ at *m/z* 401], was detected in trace amounts in CSE, being tentatively identified as naringenin-*O*-pentoside (releasing one fragment at *m/z* 269, loss of a pentosyl moiety – *m/z* 132 u). To the best of our knowledge, the presence of this compound in CS extracts was not previously reported. Peaks 7–9 and 14 were attributed to four isomers forms of aromadendrin-*O*-hexoside, based on the presence of the common fragment at *m/z* 317 (aromadendrin – H)¯. Aromadendrin derivatives have been previously evidenced in CS hydroethanolic and water extracts (Bastos et al., 2015). Compound 22 [(M–H)^¯^ at *m/z* 417] released a fragment at *m/z* 255 [(M–162)¯ corresponding to the loss of hexosyl moiety), being associated to a pinocembrin-7-*O*-glucoside. Compounds 24 and 27 [(M–H)¯ at *m/z* 447] revealed a fragmentation patter that match with either sakuranin (sakuranetin-5-*O*-glucoside) or dihydrowogonin-7-*O*-glucoside, thus it was possible to assume one of the identifications. Even though the flavanones were extracted preferentially in hydroalcoholic medium, it was observed that compounds 8, 9, and 19 were found in higher amounts in CSD than CSE.

Three isoflavones were detected, such as compounds 18 and 19, which both revealed similar characteristics and were tentatively assigned as genistein-*O*-hexoside. Nevertheless, taking into account previous findings (Bastos et al., 2015), compound 18 was identified as genistein-7-*O*-glucoside. Compound 25 [(M–H)^¯^ *m/z* 445] revealed a molecular weight 15 u higher than compounds 19 and 18, so it was assumed that it might correspond to methylgenistein-*O*-hexoside.

The presence of several flavones was also confirmed in both types of extracts. Quercetin-3-O-rutinoside (rutin), quercetin-3-O-glucose, kaempferol-3-O-rhamnoside, isorhamnetin-3-O-rhamnoside, and kaempferol-3-O-glucoside were identified in comparison with the commercial standard. Among them, rutin (quercetin-3-*O*-rutinoside) was found as the major compound (1.01 ± 0.03 mg/g in CSE and 0.404 ± 0.003 mg/g in CSD). Kaempferol-3-*O*-rhamnoside could be quantified only in CSD, while kaempferol-3-*O*-glucoside was found in both extracts. The presence of kaempferol glycosides in CS extracts was also previously reported in similar studies (Bastos et al., 2015; Aires et al., 2017; Jesus et al., 2019; Nastić et al., 2020). Only one flavonol-type compound (peak 11) was found in the extracts; it released a fragment ion at *m/z* 303 [(taxifolin – H)¯; loss of a hexosyl moiety, −162 u), being tentatively identified as taxifolin-3-*O*-glucoside.

**FIGURE 1:**
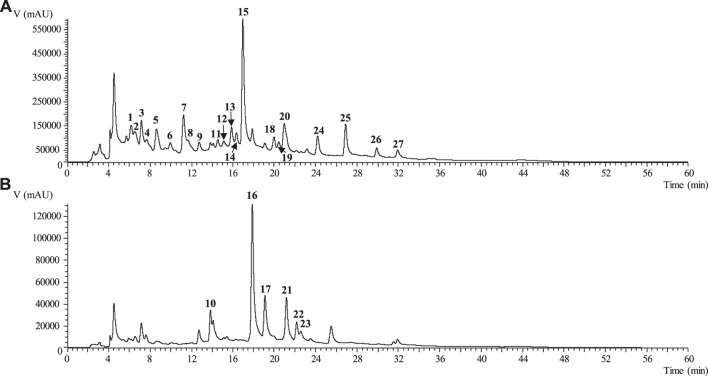
Phenolic profile of CSE recorded at 280 nm **(A)** and 370 nm **(B)**.

Overall, LC-DAD/ESI–MS^2^ analysis showed that the use of 70% ethanol as extraction solvent increased the extraction yield of phenolic compounds, CSE containing various types of phenolics in high amounts than CSD (Figure 1). The influence of extraction method and extraction solvent on phenolic compounds recovery from CS was previously studied by other authors. Nastić et al. showed that the use of dual solvent mixture can enhance the extraction efficiency, higher percentages of ethanol lead to a decrease in the extraction yield; it can be noticed that, in this study, CS extracts were obtained by pressurized liquid extraction (PLE) and supercritical fluid extraction (SFE) (Nastić et al., 2020). Aqueous extracts (infusion and decoction) represent a cheap and household alternative option to exploit the benefits of CS as source of phenolic compounds, based on ethnopharmacology recommendations.

### Preliminary Biochemical Assessment of Antioxidant Potential

#### Antioxidant Assays

The free radical scavenging activity of CSE and CSD was evaluated using the DPPH and ABTS radical scavenging assays. As shown in Table 1, the value of DPPH radical scavenging activity for CSE was 30.02 ± 0.58 mmol TE/g extract, while for CSD decreased at 14.32 ± 2.00 mmol TE/g extract; a similar trend was also observed in ABTS and FRAP assay (107.14 ± 1.43 mg TE/g extract for CSE and 55.65 ± 3.62 mg TE/g extract for CSD, respectively, 111.87 ± 4.14 mg TE/g extract for CSE and 61.07 ± 2.83 mg TE/g extract for CSD). These variations can be explained based on the major differences observed in total and individual phenolic compounds distribution in the extracts. It was previously reported that the presence of *p*-coumaric acid and the *p-*coumaroylquinic acid derivatives increased total antioxidant capacity of 70% methanolic extracts obtained from CS (tested through ABTS assay) (Aires et al., 2017). Moreover, a comparative evaluation of DPPH scavenging activity and ferric reducing ability between infusion, decoction, and hydroalcoholic extract of CS confirmed the highest antioxidant potential for the last one correlated to the higher phenolic compounds concentration found in this type of extract (Bastos et al., 2015). A significant amount of experimental data indicate that oxidative stress may contribute not only to preexisting diseases like atherosclerosis or hypertension but it may also generate oxidative damage to renal tubular cells, reducing kidney functionality (Dennis and Witting, 2017) Thus, a potential antioxidant activity could have nephroprotective effects contributing to a normal renal homeostasis, but additional studies are necessary to clarify this relation.

### Biological Activities Evaluation

#### Tyrosinase Inhibitory Capacity

Tyrosinase is a copper-containing enzyme responsible for the oxidation of tyrosine to l-DOPA and the hydroxylation of l-tyrosine, with an important role in melanin synthesis (a pigment that regulates skin color and plays a protective role by absorbing ultraviolet sunlight and removing reactive oxygen species from the skin), also responsible for browning of damaged fruits and vegetables (Rusu et al., 2018). Recent studies confirmed the link between tyrosinase inhibition and positive effects in several degenerative diseases (i.e., Parkinson), proving the importance of tyrosinase inhibitors as neuroprotective agents (Hubert et al., 2016; Tan et al., 2016). *In vitro* evaluations shown that tyrosinase can interfere with the activity of bradykinin and vasopressin, two hormones that modulate diuresis and blood pressure through renin–angiotensin–aldosteron system (RAAS) in a pH-dependent manner (BISSET, 1962).

In this regard, the evaluation of tyrosinase inhibitory capacity of CS extracts (Table 3) revealed a superior potency for CSD (IC_50_ = 3.03 ± 0.35 mg/ml) in comparison with CSE (IC_50_ = 8.66 ± 1.23 mg/ml). The obtained results cannot be correlated with the presence of phenolic compounds in the extracts, but preliminary explains that some polar and hydrophilic secondary metabolites from CS could exert a strong tyrosinase inhibitory potential. It was previously reported that cherry juice (Cásedas et al., 2016) and cherry tree bark extracts (Hubert et al., 2016) have a moderate inhibitory activity on tyrosinase. To the best of our knowledge, we evaluated for the first time tyrosinase inhibitory potential of CS extracts; our results represent a start point for further assessments of chemical composition of aqueous extracts obtained from CS and encourage supplementary evaluation of the molecular mechanisms responsible for anti-tyrosinase properties of CS.

**TABLE 3:** Enzyme inhibitory effects of the extracts of CSE and CSD (values expressed are mean ± S.D. of three parallel measurements, *p* < 0.05).

Probe ID	IC_50_ (mg/ml)
CSE	8.66 ± 1.23
CSD	3.03 ± 0.35
Kojic acid	0.05 ± 0.01

#### Diuretic Effect

**FIGURE 2:**
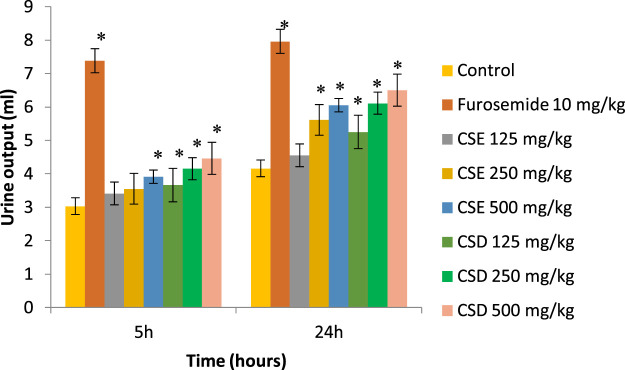
Effect of the cherry stems extract (CSE) and cherry stems decoction (CSD) on urine output recorded at 5 and 24 h in saline-loaded Crl:WI rats (*p < 0.05 vs. saline control).

As shown in Figure 2, the cherry stems extract (CSE) and cherry stems decoction (CSD) produced a dose-dependent gradual increase of the urine output, the effect being more intense at 24 h. Cherry stems decoction (CSD) at 500 mg/kg produced the most intense diuretic effect with a urine output of 6.5 ± 0.35 ml at 24 h. The reference loop diuretic, furosemide, augmented the urine output from the first hours after the oral administration, the effect increasing sharply at 5 h (7.38 ± 0.36 ml), then reaching a plateau (7.96 ± 0.37 ml), typical for a high ceiling diuretic drug. The diuretic effect of cherry stems extract (CSE) and cherry stems decoction (CSD), although inferior to furosemide, was more gradually installed, which can be of importance in a series of chronic cardiovascular diseases where a rapid diuretic effect is not desirable.

As shown in Table 4, the administration of cherry stems extract (CSE) and cherry stems decoction (CSD) increased the diuresis at 24 h in a dose-dependent manner. Rats treated with CSE showed a statistically significant increase of urine output at 250 and 500 mg/kg, while rats treated with CSD showed statistically significant results for all the three doses. The diuretic action ranged from 1.09 to 1.45 for the animals treated with CSE and from 1.26 to 1.56 for the animals treated with CSD. The strongest diuretic activity was observed in the animals treated with CSD at 500 mg/kg, which showed 81% from the activity of furosemide, the reference diuretic drug. Also, the cherry stems extract (CSE) and cherry stems decoction (CSD) increased the urinary excretion of Na^+^ and K^+^ ions (U_Na_, U_K_), the main cationic electrolytes from the urine. As shown in Table 5 the urinary excretion of the aforementioned electrolytes produced by CSE and CSD was superior to the negative control group, and presented a similar pattern with the diuretic effect, being more intense in the 5–24 h time interval.

**TABLE 4:** Parameters of the diuretic effect in saline-loaded Crl:WI rats treated with cherry stems extract (CSE) and cherry stems decoction (CSD). Values of urine output are expressed as Mean ± SD (*n* = 6).

Group (dose)	Urine output at 24 h (ml)	Diuretic action	Diuretic activity
Saline control	4.16 ± 0.37	–	–
Furosemide (10 mg/kg)	7.96 ± 0.37	1.91	1
CSE 125 mg/kg	4.55 ± 0.65	1.09	0.57
CSE 250 mg/kg	5.61 ± 0.38^a^	1.34	0.70
CSE 500 mg/kg	6.05 ± 0.33^a^	1.45	0.76
CSD 125 mg/kg	5.25 ± 0.32^a^	1.26	0.65
CSD 250 mg/kg	6.11 ± 0.21^a^	1.46	0.76
CSD 500 mg/kg	6.50 ± 0.35^a^	1.56	0.81

a *p* < 0.05 vs. saline control.

**TABLE 5:** Effect of the cherry stems extract (CSE) and cherry stems decoction (CSD) on urinary excretion of sodium (U_Na_) and potassium (U_K_) 5 and 24 h after the substance administration, fractional excretion of sodium (FE_Na_), and the ration Na/K in saline loaded Crl:WI rats.

Group (Dose)	U_Na_ at 5 h (mEq/kg)	U_K_ at 5 h (mEq/kg)	U_Na_ at 24 h (mEq/kg)	U_K_ at 24 h (mEq/kg)	Fe Na at 24 h (%)	Na/K at 24 h
Saline control	1.43 ± 0.31	1.13 ± 0.27	1.94 ± 0.37	1.55 ± 0.42	1.32	1.25
Furosemide (10 mg/kg)	5.59 ± 0.78*	4.91 ± 0.41*	6.32 ± 0.84*	5.31 ± 0.44*	6.81	1.19
CSE (125 mg/kg)	1.83 ± 0.35	1.57 ± 0.29	2.58 ± 0.61	1.96 ± 0.59	1.97	1.31
CSE (250 mg/kg)	2.41 ± 0.66*	1.83 ± 0.31*	2.88 ± 0.59*	2.01 ± 0.73*	2.14	1.43
CSE (500 mg/kg)	2.89 ± 0.75*	2.02 ± 0.89*	3.03 ± 0.88*	2.14 ± 0.69*	2.42	1.41
CSD (125 mg/kg)	2.15 ± 0.39	1.83 ± 0.73	2.87 ± 0.83	2.08 ± 0.68	2.05	1.37
CSD (250 mg/kg)	2.49 ± 0.44	2.12 ± 0.29	3.42 ± 0.87	2.39 ± 0.46	3.72	1.43
CSD (500 mg/kg)	3.11 ± 0.82	2.36 ± 0.55	3.84 ± 0.64	2.71 ± 0.32	4.58	1.41

Values of U_Na_V and U_K_V are expressed as Mean ± SD (*n* = 6) (*p < 0.05 vs. saline control).

The most significant excretion of the tested electrolytes was produced by the 500 mg/kg dose of CSD with U_Na_ and U_K_ values of 3.84 ± 0.64 and 2.71 ± 0.32 mEq/kg, 24 h after the substance administration. The calculated Na^+^/K^+^ ratio for CSE and CSD treated groups did not show values above 10 at any moment of determination, thus indicating a lack of a potassium-sparing effect, similar with furosemide. On the contrary, both CSE and CSD produced a clear kaliuretic effect. In our experiment, the fractional excretion of sodium ions (FE_Na_), defined as the percentage of sodium ions filtered by the kidneys and not reabsorbed, was calculated at 24 h. The fractional excretion of sodium ions (FE_Na_) is a valuable parameter which can provide additional information on the tubular function, although the glomerular filtration rate and daily intake of sodium could also influence its values (Schreuder et al., 2017). As expected, the experiment showed a net increase of FE_Na_ (6.81%) for the reference diuretic drug furosemide, which specifically inhibits the sodium–potassium–chloride symporter in the thick ascending limb of the loop of Henle, and a moderate increase of FE_Na_ (2.14–2.42 and 3.72–4.58%) for CSE and CSD at doses of 250 and 500 mg/kg, also suggesting a tubular mechanism of action responsible for the diuretic effect of the tested cherry stems preparations.

The diuretic activity of powdered cherry stems (administered as capsules at an equivalent dose of 2.0 g of the plant per person) was previously evaluated in healthy human subjects, urinary volume, urinary electrolyte concentration (sodium, potassium, chloride, and calcium), and the osmolality being monitored (Hooman et al., 2009). The study revealed a mild diuretic effect for CS and a slight increase for urinary excretion of calcium, sodium, and chloride, concluding that the drug can be used as diuretic agent, but with precautious in those patients, and also in patients having any disorders associated with calcium, sodium, and/or chloride deficiency (especially in those with urolithiasis because of rising calcium excretion). A comparison between those results and our experimentally output regarding diuretic activity of CS reveals a similar trend for both type of tested CS-derived products. Our study represents the first report about diuretic and saluretic activity of the extracts obtained from CS and correlates this potential with the presence of phenolic secondary metabolites as main constituents of both hydroalcoholic and aqueous preparations.

## Conclusion

Based on ethnopharmacological evidence, cherry stems (CS) preparations (hydroalcoholic extract and decoction) were evaluated for their phenolic profile and several bioactivities. Overall, the presence of phenolic secondary metabolites was confirmed in both extracts, the highest concentrations being found in hydroalcoholic extract. Flavonoids were the main type of phenolic compounds identified in the extracts, naringenin derivatives being quantified in high amounts. Hydroalcoholic extract exerted an important antioxidant activity, while tyrosinase inhibitory potential and diuretic activity were superior for decoction.

Our findings suggest that cherry stems are a valuable and less exploited by-product rich in phenolic secondary metabolites, with potential applications as a mild and safe diuretic agent. Even through traditional medicine recommends the use of aqueous preparations obtained from CS as adjuvant therapy in urinary tract disorders, hydroalcoholic extracts could represent an improved alternative, showing a similar diuretic potential. Nevertheless, future investigations need to be performed in order to elucidate the intimate mechanisms responsible for CS pharmacological potential.

## Data Availability

The original contributions presented in the study are included in the article/Supplementary Material, further inquiries can be directed to the corresponding author.
